# Editorial: Burnout in the health, social care and beyond: Integrating individuals and systems

**DOI:** 10.3389/fpsyt.2023.1166060

**Published:** 2023-03-01

**Authors:** Simon Surguladze, Antigonos Sochos, Eka D. Chkonia, Anthony J. Montgomery

**Affiliations:** ^1^South London and Maudsley National Health Service Foundation Trust, London, United Kingdom; ^2^School of Psychology, University of Bedfordshire, Luton, United Kingdom; ^3^Department of Psychiatry, Tbilisi State Medical University, Tbilisi, Georgia; ^4^Department of Psychology, Northumbria University, Newcastle upon Tyne, United Kingdom

**Keywords:** burnout—professional, care systems, remediation, vulnerabilities, cognition

This Research Topic aimed at widening our knowledge base on occupational burnout, with the emphasis on interdisciplinary studies. The Topic includes 13 papers from various fields (e.g., health services, service and hospitality sectors and education), that looked at links between individual vulnerabilities, systemic failures and burnout.

## Systems and individuals

Three papers explored an interplay between systems and individuals by using Job Demands and Resources (JD-R) model.

Zang and Chen looked at the relationship between person-organization fit, job satisfaction and teacher burnout in kindergartens in China and found that these three variables were negatively correlated. The authors further demonstrated that compared with job resources, job demand was more significantly correlated with burnout, that is, individuals were more likely to suffer from burnout when job failed to meet their needs. A study of Deng et al. reported opposite results, that probably was due to differences in sample characteristics. This study investigated burnout in employees of non-profit sector in China. The study found that job resources (JR) and job demands (JD) had opposite effects on burnout and psychological distress. The differential magnitude of the estimates suggested that JR had a greater effect on burnout compared with the effect of JD. Based on this the authors emphasized the importance of JR (e.g., positive relationships with colleagues and/or supervisors or availability of job-related information) as potentially protective factor. The lack of JR could be more detrimental to the health of employees than high degrees of JD, e.g., workload, emotional workload, and changes in tasks.

Wójcik et al. investigated burnout in nurses in Poland during the late wave of COVID-19 pandemic. Apart from the job demands and resources (JD-R) exploration, the researchers were interested in such variables as interpersonal conflict and coping modes (deep coping vs. surface coping). The study found significant positive associations between organizational constraints, interpersonal conflict at work and burnout among nurses. Importantly, higher organizational constraints and conflicts at work were found to be associated with an increase in surface acting, which was in turn associated with an increase in burnout. The authors advocated for strengthening organizational support and adaptive coping strategies as potential factors to alleviate burnout. This was particularly important in the context of COVID-19 pandemic, during which many nurses perceived their workplace as potentially “harmful and dangerous”.

A *Perspective* article of Kakarala and Prigerson reviewed inadequacies of US medical system that underlie risks of burnout and high suicide risk of medical professionals. The authors listed a number of factors that in their view contribute to physicians' burnout and suicide risk in US, e.g., documentation burden, declining professional autonomy, lack of confidentiality, work pressures imposed by insurance companies and financial incentives to increase revenue while cutting costs. On the other hand, mental health stigma and intrusive medical licensing applications remain barriers to physicians seeking help, which compound physicians' work stress. The authors stated that COVID-19 has laid bare a longstanding problem: the US. medical system undermines physicians' needs while restricting their autonomy and options for self-help. The authors suggest that more research, especially comparative studies with other countries are needed to find the ways to “detoxify” US medical system.

## Remediation programs

The paper by Appelbom et al. highlights the benefits of the long-term allocation of resources and establishment of relevant procedures toward the provision of psychological support to health care staff, during the COVID-19 pandemic. The authors report on the implementation of a psychological support model that at an intensive care unit in Stockholm, Sweden, during the first wave of the pandemic. The initiative aimed at promoting resilience among frontline staff and included education and training, peer support, group and individual sessions, on-boarding for transferred staff, and manager support. Findings suggested that the most effective components of the provision were peer support and daily group sessions, but only when these are structurally integrated in clinical practice.

In line with the rapid expansion of online technologies, a workplace web-based blended psychoeducation randomized controlled trial was offered to staff of industries that were considered to have a high level of work-related stress, such as the service and hospitality sectors (Lam et al.).

The program aimed at enhancing the mental wellbeing and mental health literacy of workers in the workplace. It comprised two main components, an individual-directed psychoeducation course, and an organization-directed consultation. This psychoeducation course is a blended program, using the e-Learning approach, followed by a face-to-face group session at the end of the course. Comparisons of the outcome between the intervention and control groups were statistically significant in favor of the intervention group on most measures, eg 3 out of 4 measures of Mental health literacy, one (professional accomplishment) out of 3 measures of burnout and the measure if stress. It should be noted that the results reflected just a short -term (immediate) outcome, whereas the longer-term outcome is yet to be determined.

## Underlying factors

Yan et al. highlighted a sex effect on vulnerability to burnout in a sample of dental post-graduates. The authors found that the prevalence of job burnout, career choice regret and depressive symptoms were higher in females compared with males. The career choice regret is experienced when the obtained career is not what the student expected or hoped for. The study indicated that career choice regret had a stronger association with burnout in female graduates but not in males. The authors suggested that career choice regret could induce an aversion to the chosen career, thereby increasing the risk of job burnout.

Sampei et al. investigated how the risk of emotional exhaustion was associated with mindfulness skills and social support in a single medical center in Japan. In their one-hospital study, the authors found that the factors associated with emotional exhaustion differed by whether the worker had high exposure to SARS-CoV-2. For example, higher social support was associated with a reduced odds of emotional exhaustion only among the highly exposed group. Among those highly exposed, participants with a lower level of mindfulness, compared to those with a higher level, had significantly higher odds of emotional exhaustion.

Montgomery and Lainidi explore another interesting factor potentially underlying healthcare workers' burnout vulnerability—employee silence. Reviewing the relevant literature, the authors find evidence that organizations which discourage staff from speaking up and reporting concerns, face relatively high staff burnout and compromise patient safety and quality of care. The evidence also points out that a professional culture of employee silence starts developing in the first years of medical education and is maintained after graduation and thought one's career. The authors emphasize the role of management in sustaining or challenging an organizational culture of silence and advocate the adoption of compassionate leadership, valuing openness and sharing.

## Consequences of burnout

Two papers by Koutsimani and Montgomery (b) address the effect of burnout on cognitive functioning. In a longitudinal study aiming to clarify the direction of causality between non-clinical burnout and cognitive function as well as between burnout, depression, and anxiety, the authors did not find compelling evidence for the negative effects of burnout on cognitive capacity. However, they did find that cynicism, rather than emotional exhaustion, had a negative impact on visuospatial abilities while a high sense of personal efficacy showed mutual associations with stronger executive functions. Moreover, burnout was different from but reciprocally associated with anxiety and depression. The findings of the study also highlight the role of perceived family support as protective against burnout and cognitive dysfunction. To explore further the impact of burnout on the relatively neglected area visuospatial functioning, the authors conducted a mini review of studies involving health professionals [Koutsimani and Montgomery (a)].

The evidence reviewed, appeared too sparse to allow clear conclusions, but it highlighted the need for further studies in this important area.

Alameri et al. looked at the associations between burnout and cardiovascular risk. The risk was measured by Fuster-BEWAT tool that included 5 main variables: blood pressure, exercise, weight, diet, and tobacco (https://www.sciencedirect.com/science/article/pii/S0735109715070965?via%3Dihub).

The authors reported that burnout and emotional exhaustion were associated with an elevated cardiovascular risk. Further, the model showed a positive association between personal accomplishment and cardiovascular health. Due to cross-sectional nature of the study, causality could not be determined.

High levels of burnout and job dissatisfaction have been commonly observed amongst General Practitioners (GPs). This could be related to diagnostic uncertainty of the community cases.

Zhou et al. examined the relationship between burnout and uncertainty among 70 general practices in England (randomly selected). Almost one-third of GPs reported experiencing >10% of diagnostic uncertainty in their day-to-day practice over the past year, greater diagnostic uncertainty had higher levels of emotional exhaustion, job dissatisfaction and turnover intentions.

## Concluding remarks

The 13 papers in this Research Topic add to the growing evidence that the antecedents of burnout are rooted in the job demands and resources (and lack of fit) within the organizations studied. Future research needs to address the combined impact of individual (e.g., cognitive functioning, career regret), interpersonal (e.g., colleague relations) and organizational (e.g., administrative demands, financial targets) factors in designing interventions that prevent the development of burnout. Interventions need to be appropriately embedded in organizations, not *ad-hoc*, and part of a wider strategy of developing healthy workplaces. Burnout is symptom of organizational dysfunction, a starting point not an end point.

## Author contributions

All authors listed have made a substantial, direct, and intellectual contribution to the work and approved it for publication.

